# Cartilage-Specific Ablation of Site-1 Protease in Mice Results in the Endoplasmic Reticulum Entrapment of Type IIB Procollagen and Down-Regulation of Cholesterol and Lipid Homeostasis

**DOI:** 10.1371/journal.pone.0105674

**Published:** 2014-08-22

**Authors:** Debabrata Patra, Elizabeth DeLassus, Guosheng Liang, Linda J. Sandell

**Affiliations:** 1 Department of Orthopaedic Surgery, Washington University School of Medicine, St. Louis, Missouri, United States of America; 2 Department of Cell Biology and Physiology, Washington University School of Medicine, St. Louis, Missouri, United States of America; 3 Department of Molecular Genetics, University of Texas Southwestern Medical Center, Dallas, Texas, United States of America; University of Rochester, United States of America

## Abstract

The proprotein convertase site-1 protease (S1P) converts latent ER-membrane bound transcription factors SREBPs and ATF6 to their active forms. SREBPs are involved in cholesterol and fatty acid homeostasis whereas ATF6 is involved in unfolded protein response pathways (UPR). Cartilage-specific ablation of S1P in mice (S1P*^cko^*) results in abnormal cartilage devoid of type II collagen protein (Col II). S1P*^cko^* mice also lack endochondral bone development. To analyze S1P*^cko^* cartilage we performed double-labeled immunofluorescence studies for matrix proteins that demonstrated that type IIB procollagen is trapped inside the ER in S1P*^cko^* chondrocytes. This retention is specific to type IIB procollagen; other cartilage proteins such as type IIA procollagen, cartilage oligomeric matrix protein (COMP) and aggrecan are not affected. The S1P*^cko^* cartilage thus exhibits COMP-, aggrecan-, and type IIA procollagen-derived matrices but is characterized by the absence of a type IIB procollagen-derived matrix. To understand the molecular reason behind S1P*^cko^* phenotypes we performed genome-wide transcriptional profiling of cartilage isolated from S1P*^cko^* and wild type littermates. While the UPR pathways are unaffected, the SREBPs-directed cholesterol and fatty acid pathways are significantly down-regulated in S1P*^cko^* chondrocytes, with maximal down-regulation of the stearoyl-CoA desaturase-1 (Scd1) gene. However, mouse models that lack Scd1 or exhibit reduction in lipid homeostasis do not suffer from the ER retention of Col II or lack endochondral bone. These studies indicate an indispensable role for S1P in type IIB procollagen trafficking from the ER. This role appears not to be related to lipid pathways or other current known functions of S1P and is likely dependent on additional, yet unknown, S1P substrates in chondrocytes.

## Introduction

Site-1 protease (S1P; also called the membrane-bound transcription factor protease, site-1) is a proprotein convertase that converts latent, endoplasmic reticulum (ER) membrane-bound transcription factors into their free and active form. Two developmental pathways regulated by S1P that have been studied extensively include cholesterol and fatty acid homeostasis and the unfolded protein response [1]. During cholesterol and fatty acid homeostasis, S1P plays a fundamental role in the processing of the transcription factors sterol regulatory element binding proteins (SREBP-1a, -1c, and -2) [2]. During unfolded protein response (UPR), S1P plays a critical role in the processing of activating transcription factor 6 (ATF6) [3], old astrocyte specifically induced substance (OASIS) [4], and the cAMP-responsive element binding protein H (CREBH) [5]. All these pathways are fundamental in maintaining cellular homeostasis and therefore S1P plays major and critical roles in fundamental developmental pathways. Mutational inactivation of S1P in zebrafish (*gonzo*) results in abnormal lipid metabolism and skeletal formation [6].

Mammalian skeletal development begins early during embryogenesis. It involves endochondral bone formation, a process by which most long and axial bones of the body are formed [7]. During this process, mesenchymal cells aggregate to form condensations and cells in the center of these condensations differentiate to form chondrocytes [8]. Chondrocytes secrete a specialized extracellular matrix (ECM) called cartilage that forms the anlagen for future skeleton, whereas the surrounding undifferentiated cells form the perichondrium [9]. The innermost cells within the chondrocytes differentiate to form hypertrophic chondrocytes, with cells in the inner layer of the perichondrium differentiating into bone-forming osteoblasts resulting in a bone collar around the cartilaginous core [10]–[12]. Fully differentiated hypertrophic cells secrete a distinct ECM which gradually becomes calcified before they die through apoptosis. The mineralization of the ECM allows for vascular invasion from the bone collar and the entry of osteoclasts and osteoblasts that degrade the mineralized cartilage to deposit endochondral bone [13]. Deposition of a type I collagen-rich bone matrix results in two opposing growth plates allowing for bone growth in both directions [14]. In contrast to endochondral bone formation, much of the craniofacial skeletal bones are formed by intramembranous ossification where the mesenchymal cells differentiate directly into osteoblasts without requiring a cartilage intermediate.

It follows from this developmental paradigm that factors that affect cartilage development also impact endochondral bone development. The link of S1P to cartilage development was shown by the study of zebrafish *gonzo* mutant [6]. In a previous study we showed that S1P is required for proper cartilage matrix development in mice [15]. By creating cartilage-specific S1P knockout mice (S1P*^cko^*) we demonstrated that these mice do not form any endochondral bone. S1P*^cko^* mice also exhibited poor cartilage development with most of the type II collagen protein (Col II) trapped inside the cell, resulting in a drastic reduction of Col II in the cartilage. Ultrastructural analysis of the cartilage showed engorged and fragmented ER.

In the current study we investigated the nature of Col II entrapment and the mechanistic reasons behind S1P*^cko^* phenotypes. Lack of S1P would result in lack of activation of SREBPs, ATF6, OASIS, and CREBH. Therefore, lack of S1P activity in chondrocytes would be expected to affect both the SREBPs-directed cholesterol and fatty acid homoeostasis and the UPR pathways. In order to understand how lack of S1P affects the downstream pathways, transcriptional profiling in chondrocytes was performed by genome-wide expression analyses with RNA extracted from the cartilage of S1P*^cko^* and wild type (WT) littermates. Our studies show that the SREBPs-dependent cholesterol and fatty acid biosynthetic pathways are down-regulated in S1P*^cko^* chondrocytes. In contrast, UPR pathways remain unaffected. Furthermore, lack of S1P in cartilage results specifically in the ER retention of type IIB procollagen (pro-Col IIB). These data suggest that S1P has an indispensable function in pro-Col IIB trafficking from the ER to the cartilage matrix. However, our in depth mechanistic analyses indicate that this activity is not related to current known functions of S1P. Additional, yet unidentified, S1P substrates presumably modulate Col II trafficking from the ER to the cartilage ECM.

## Materials and Methods

### Ethics Statement

All mouse procedures were performed in accordance with National Institutes of Health's Guide for the Care and Use of Laboratory Animals using vertebrate animals/ethics protocols reviewed and approved by the Animal Studies Committee at Washington University School of Medicine.

### Double-labeled immunofluorescence studies

Double-labeled immunofluorescence to analyze retention of matrix proteins in the ER were performed on 5- µm formalin fixed, paraffin embedded sections from E16.5 embryonic tissues processed as described [15]. Following rehydration through graded ethanol solutions, the sections were washed in phosphate buffered saline, pH 7.4 (PBS) for 10 min. For antigen retrieval the slides were treated with Proteinase K (10 µg/mL in 10 mM Tris-HCl pH 7.4) for 30 min at 37°C followed by blocking for 1 h in either 10% donkey serum or 10% goat serum. The two primary antibodies (Table 1) were added together in 1.5% donkey serum (or goat serum) and incubated in a humidified chamber for 1 h. The slides were washed three times in PBS for 15 min and rinsed once in distilled water. The two secondary antibodies were then added together and incubated for 1 h, washed thoroughly and mounted with Vectashield DAPI (Vector Laboratories, Inc., CA). Antibody concentrations were as follows: antibodies to Col II triple helical domain (THD; IIF antibody [16]), Col IIA (designed against exon 2; IIA antibody) [15], [16], aggrecan (rabbit antisera against rat aggrecan, a gift of Dr. Kurt Doege), and cartilage oligomeric matrix protein (COMP) [17] were used at 1∶100. Affinity-purified IIBN antibody (designed against type II collagen exon 1–3 peptide junction and specific to type IIB procollagen) [18] was used at 5 µg/mL, while goat anti-human calnexin (Santacruz Biotechnology) and anti-XBP-1 (Biolegend, CA, USA) antibodies were used at 1∶50. The secondary donkey anti-goat Alexa 488 (Invitrogen) and donkey anti-rat Alexa 594 (Invitrogen) antibodies were all used at 1∶250. Images were captured using a 60X, 1.4 NA oil immersion objective mounted on an Eclipse E800 microscope (Nikon) and QImaging Retiga 2000R Fast 1394 camera and deconvolved. For deconvolution imaging, MetaMorph software (Molecular Devices) was used to control the Z-motor device (Prior Scientific), to capture and deconvolve images, and to compile them to give a final image. All images shown are for mature columnar proliferative chondrocytes in the WT and for a corresponding region in S1P*^cko^* (Fig. S1A).

**Table 1:** Summary of antibodies used for immunohistochemical analyses.

Antibody	Protein recognized
IIF	Type II collagen triple helical domain (THD)
IIA	Type IIA procollagen (Col IIA)
IIBN	Type IIB procollagen (pro-Col IIB)
Anti-calnexin	Calnexin
Anti-XBP-1	XBP-1
Anti-aggrecan	Aggrecan
Anti-COMP	COMP

### Genome-wide expression profiling

To profile gene expression in S1P*^cko^* cartilage and to contrast it with WT, we harvested the chondroepiphyseal cartilage from the tips of long bones in forelimbs/hindlimbs (Fig. S1B) of freshly harvested embryonic (E) 16.5 S1P*^cko^* (S1P*^f/f^*;Col2-Cre) embryos and its WT (S1P*^f/f^*, S1P*^f/+^*, or S1P*^f/+^*;Col2-Cre) littermates, as the mutant mice die during or very soon after birth. At E16.5 the mutant is easily distinguished without genotyping, plus identifying and separating the bones from the musculature is easier than at later time points in the mutant. After harvesting the bones, excess muscle tissue and blood vessels were removed by rolling on Whatman filter paper, and the chondroepiphyseal cartilage from the ends of the bones were broken off (Fig. S1B) and stored in Trizol (Invitrogen) at −80°C for RNA extractions later. In some cases the chondroepiphyseal cartilage was not separated and the entire long bones kept intact and stored in Trizol for RNA extraction. The calvarium was also surgically removed in some cases and stored in Trizol at −80°C and RNA extracted as for the chondroepiphysis. Therefore three kinds of tissue were harvested: chondroepiphyseal cartilage from the tips of long bones, entire long bones (includes both the bone and epiphyseal cartilage), and calvaria.

To extract RNA, the harvested tissues were first homogenized in Trizol using a polytron and RNA extracted according to the manufacturer's recommended protocol and column purified by RNeasy mini kit (Qiagen). Genome-wide expression profiling was performed using Illumina MouseWG-6 v1.1 Expression BeadChip which profiles 45,200 mouse transcripts. Statistically significant differentially expressed genes (false discovery rate (FDR) of 5%) in S1P*^cko^* chondrocytes as compared to WT were identified by 2-way ANOVA (analysis of variation) analysis using Partek Genomics Suite (St. Louis, MO). Only genes that were two-fold or more differentially regulated with significant *p*-values (*p*<0.05) were carried forward for characterization. Differential gene expression data derived from microarray analysis was used to identify functional and molecular networks through the use of MetaCore GeneGo network building tools (GeneGo Inc., Carlsbad, CA). Again, only genes that were two-fold or more differentially regulated were used to generate a network score of negative log of the *p* value to determine biological pathways disrupted in S1P*^cko^* chondrocytes. The raw microarray data have been deposited in the Gene Expression Omnibus (GEO). The accession number is GSE55577 (online at http://www.ncbi.nlm.nih.gov/projects/geo).

### Quantitative real-time PCR (qPCR) analysis

Quantitative real time PCR (qPCR) was performed for confirmation of expression profiling for genes involved in cholesterol and fatty acid homeostasis. For qPCR analysis, the RNA was harvested and pooled from the chondroepiphyseal cartilage of forelimbs and hindlimbs of five E16.5 S1P*^cko^* and five E16.5 WT (three S1P*^f/f^*, one S1P*^f/+^*, and one S1P*^f/+^*;Col2-Cre mice) littermates. The RNA was extracted with Trizol and column purified as described above and used for qPCR analysis using SYBR Green primer sets, 2× SYBR Green mix (Life Technologies/Applied Biosystems) and standard protocols. SYBR Green primer sets for the murine cyclophilin, insulin-induced gene-1 (Insig-1), 3-hydroxy-3-methylglutaryl (HMG)-CoA reductase (Hmgcr/Hmdh), HMG CoA synthase (Hmgcs), farnesyl diphosphate synthase (Fdps), low density lipoprotein receptor (Ldlr), stearoyl-CoA desaturase-1 (Scd1), and Scd2 genes have been reported previously [19], [20]. Additional primer sets for the murine glyceraldehyde 3-phosphate dehydrogenase (Gapdh), fatty acid desaturase-2 (Fads2), sterol-C4-methyl oxidase-like (Sc4mol), star-related lipid transfer (START) domain containing 4 (Stard4), proteoglycan 4 (Prg4), loricrin (Lor), and fibroblast growth factor receptor 1 oncogene partner 2 (Fgfr1op2) genes were taken from the MGH/Harvard Medical School primer bank (http://pga.mgh.harvard.edu/primerbank/) and are given in Table S1. The relative amount of all mRNAs was calculated using the comparative C_t_ method with cyclophilin or Gapdh as the invariant control.

### Analysis of ER stress response in S1P^cko^ chondrocytes

RNA extracted from the chondroepiphysis of E16.5 WT and S1P*^cko^* embryos was used to further validate the unaffected UPR discerned from the microarray analysis. For X-box binding protein-1 (XBP-1) mRNA splicing assay, RNA was converted to cDNA using routine protocols and splicing analyzed by PCR or by restriction digestion. For PCR analysis, the cDNA was amplified using XBP-1 primers as described [21] and the PCR products directly analyzed in a 4.8% polyacrylamide gel to separate the products derived from the spliced and un-spliced XBP-1 mRNA based on size. Analysis by restriction digestion was performed as described [22]. Briefly, the cDNA was first amplified using the XBP-1 primers 3S and 12AS followed by digestion of the amplified PCR product with *Pst*I to selectively cleave the cDNA derived from un-spliced XBP-1 mRNA, and analyzed in a 2% agarose gel.

For a global analysis of ER stress response in S1P*^cko^* chondrocytes the RT^2^ Profiler PCR Array System for the murine UPR (Cat. No. PAMM-089C-2, SABiosciences, Qiagen), which profiles the expression of 84 key genes involved in recognizing and responding to unfolded protein accumulation in the ER, was used (a complete list of proteins profiled can be found at http://sabiosciences.com/rt_pcr_product/HTML/PAMM-089A.html) (also listed in Table S2). The RNA was converted to cDNA using the RT^2^ First Strand kit (Cat. No. C-03) and qPCR reactions set up using the RT^2^ SYBR Green/ROX qPCR master mix (Cat. No. PA-012) in a 96-well array plate following instructions recommended by the manufacturer. A total of four qPCR arrays were used, two for WT and two for S1P*^cko^* RNA for biological duplicates. Each WT or S1P*^cko^* RNA was pooled from the chondroepiphyseal cartilage of two E16.5 WT or two E16.5 S1P*^cko^* embryos, respectively. Thus a total of four WT and four S1P*^cko^* E16.5 embryos were profiled. Analysis of the data was done using the Qiagen-SABiosciences' web-based PCR data analysis software.

### Miscellaneous

S1P*^cko^* mice were generated by mating S1P*^f/f^* mice with S1P*^f/+^*; Col2-cre mice as described [15]. *Scd1*−/− mice were generated by mating *Scd1*+/− heterozygotes and identified by genotyping [23]. *Scd1*+/− heterozygote parental strains and the S1P*^f/f^* and S1P*^f/+^*;Col2-Cre strains were all fed *ad libitum* with standard laboratory chow. Analysis of endochondral bone formation in *Scd1*−/− mice by Safranin O, Fast green, and hematoxylin staining and Col II deposition by double-labeled immunofluorescence were done as for S1P*^cko^* mice at E15.5. In situ hybridization analyses were done on 5- µm paraffin-embedded sections as described previously using ^35^S-labeled riboprobes [15], [24]. Riboprobes for the murine Scd1, Scd2, Ldlr, Fads2, and Fdps genes were derived from their full-length cDNA clones purchased from commercial sources. Murine Scd1 immunohistochemistry (IHC) was performed on 5- µm paraffin-embedded sections using anti-Scd1 antibody (Santacruz Biotechnology) and HRP-conjugated anti-goat IgG and DAB. Hematoxylin was used as the counter stain. In situ hybridization images were viewed with BX51 (Olympus) microscope and images captured with a digital camera (DP70; Olympus) using DP controller software (Olympus). Images of hybridization signals were artificially colored red and superimposed on toluidine blue-counterstained images using Photoshop (Adobe).

## Results

### Retention of cartilage matrix proteins in the ER

**Figure 1:**
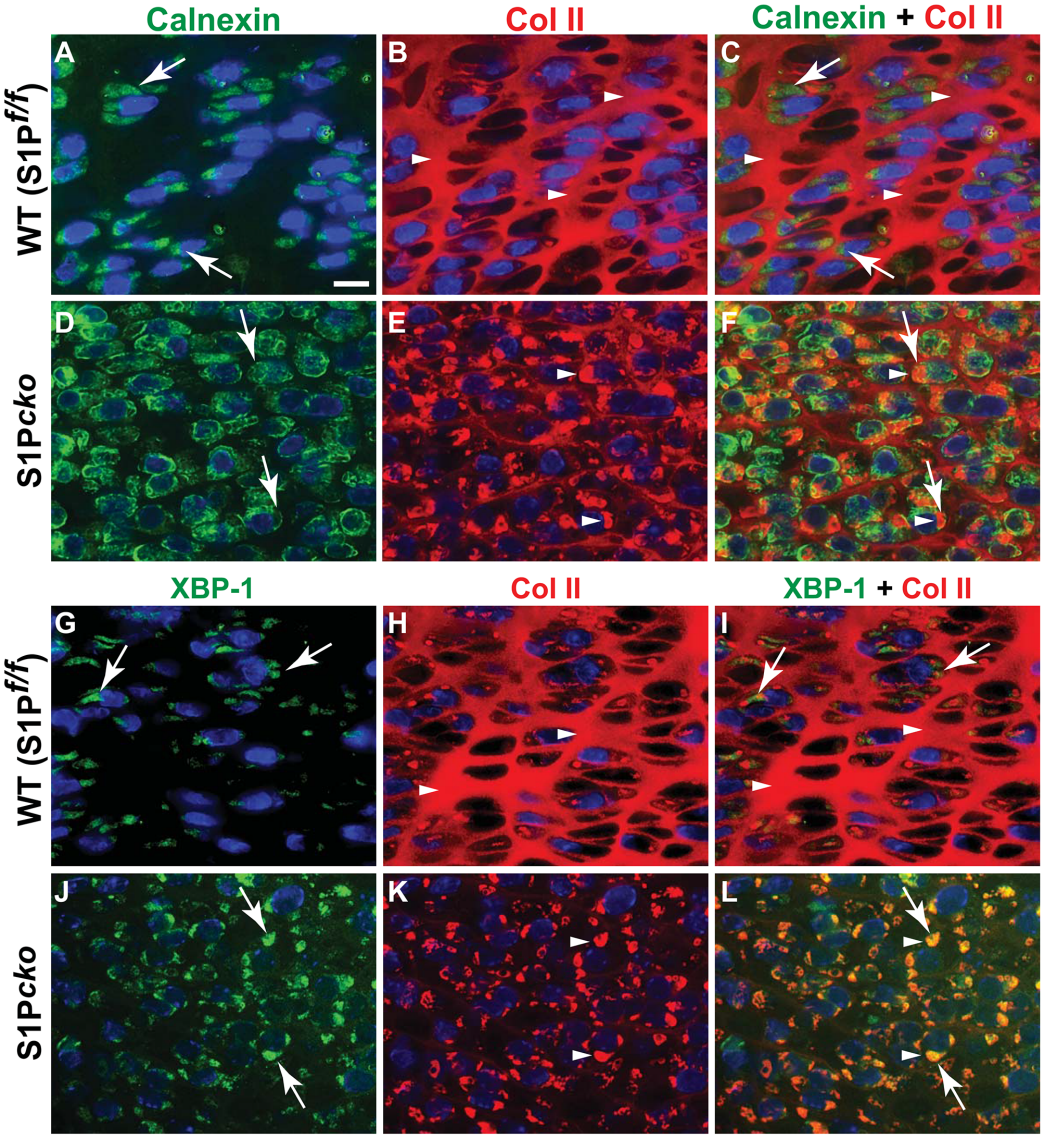
The ER retention of Col II in S1P*^cko^* chondrocytes. (A–F) Double-labeled immunofluorescence analyses for Col II THD (IIF antibody) and calnexin in E16.5 femurs in WT (**A–C**) and S1P*^cko^* (**D–F**). Signals from calnexin (**A, D**) are shown in green (white arrows) and those from Col II THD (**B, E**) are shown in red (white arrowheads) with composite signals shown in **C** and **F**. (**G–L**) Double-labeled immunofluorescence analyses for Col II THD and XBP-1 in E16.5 femurs in WT (**G–I**) and S1P*^cko^* (**J-L**). Individual signals from XBP-1 (**G, J**) are shown in green (white arrows) and those from Col II THD (**H, K**) are shown in red (white arrowheads), with composite signals shown in **I** and **L**. All images shown are for mature columnar proliferative chondrocytes in the WT and a corresponding region in S1P*^cko^* (see Fig. S1A). Bar (all panels): 10 µm.

Chondrocytes by nature are secretory cells and secrete large amounts of ECM proteins needed to create the cartilage matrix. Ultrastructural analysis of cartilage from S1P*^cko^* mice showed an enlarged ER with the retention of a crystalline material [15]. Therefore we first examined the composition of proteins retained within the ER. Using double-labeled immunofluorescence, an antibody to calnexin (an ER membrane protein) and an antibody (IIF; see Table 1) against the Col II triple helical domain (THD), we analyzed whether Col II in the mutant cartilage was trapped in the ER (Fig. 1).

In WT, the Col II THD in the cartilage (shown as red immunofluorescence, white arrowheads, Fig. 1B, C) is distinct from calnexin (shown as green immunofluorescence, Fig. 1A, C) of the ER membrane surrounding the blue DAPI-stained nuclei of the chondrocytes (Fig. 1A–C). Notice the strong presence of a Col II-derived matrix in the cartilage of the WT (Fig 1B, C). In S1P*^cko^* however, signaling from Col II THD (red) is visible primarily from inside the cells (white arrowheads, Fig. 1E) concomitant with the drastic drop of Col II-derived signals from the S1P*^cko^* cartilage. The chondrocytes appear distended from the entrapped Col II protein. In the composite image (Fig. 1F), Col II is visible as yellowish-orange immunofluorescence signal surrounding the blue-DAPI-stained nuclei, due to its colocalization with calnexin (white arrows, Fig. 1D, F) in the ER. To confirm the ER-entrapment of Col II in the mutant, we analyzed whether Col II colocalized with XBP-1 [25], a known ER luminal protein (Fig. 1G–L) in S1P*^cko^* cartilage. As shown in Fig. 1J-L, Col II colocalized with XBP-1 in the mutant chondrocytes confirming its entrapment in the ER. Even though results are shown for the proliferative zone, Col II retention is seen throughout all zones of the growth plate and is not limited by cell size, morphology or location in the growth plate. These data indicate that the matrix in S1P*^cko^* cartilage is defective and devoid of Col II due to disruption of Col II trafficking from ER to the cartilage.

**Figure 2:**
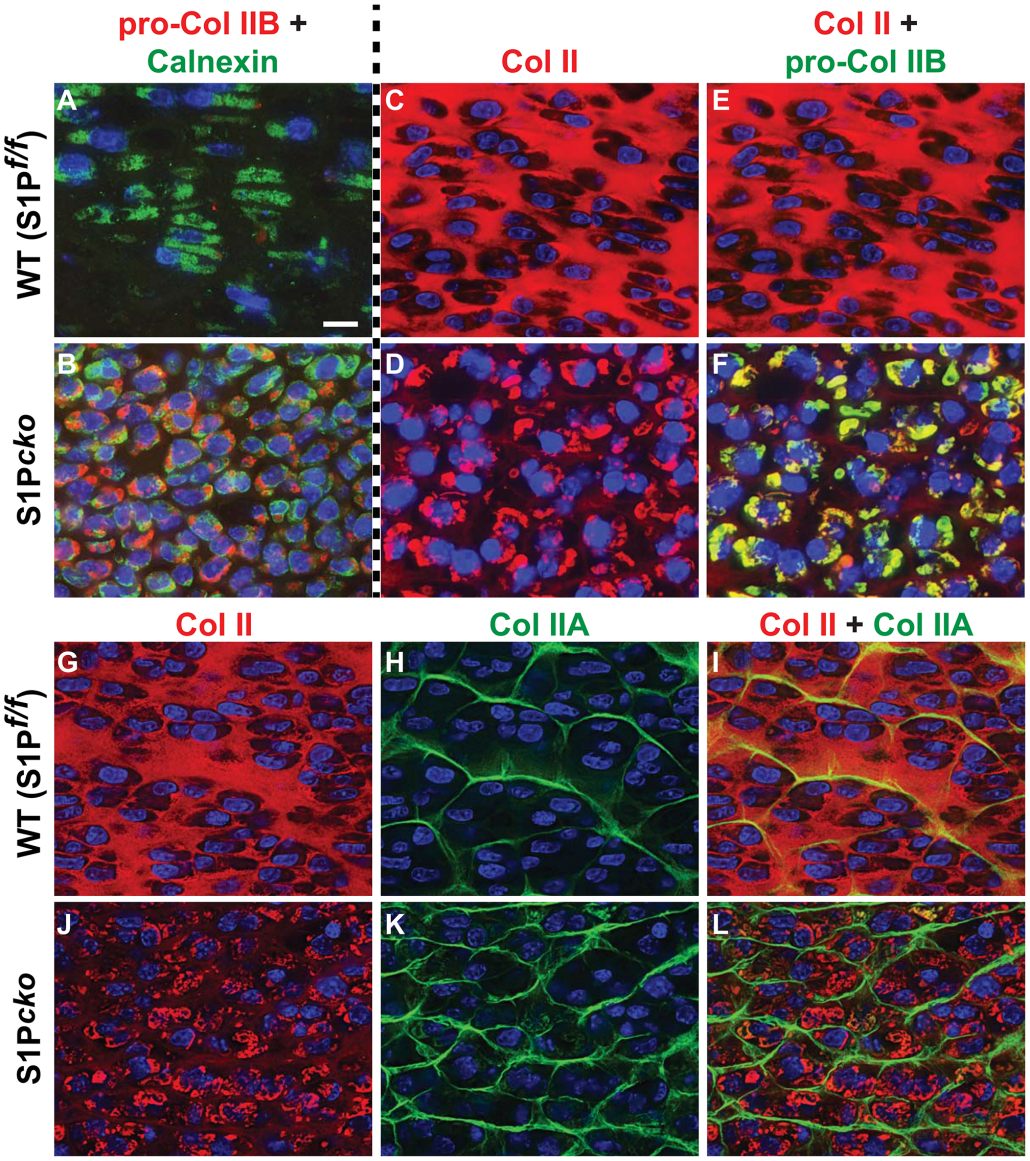
Specific retention of pro-Col IIB in S1P*^cko^* chondrocytes. Double-labeled immunofluorescence analyses were done for pro-Col IIB (red, IIBN antibody) and calnexin (green) in E16.5 femurs in WT (A) and S1P*^cko^* (B) and only the composite for this analysis is shown. In a separate analysis (symbolized by the dashed line between panels A/B and C/D), double-labeled immunofluorescence was done for Col II THD (red) (**C–F**) and pro-Col IIB (green) (**E, F**) in E16.5 femurs in WT (**C, E**) and S1P*^cko^* (**D, F**) with composite signals shown in **E** and **F**. Panels **G-L** show double-labeled immunofluorescence analyses for Col II THD (red) (**G, J**) and Col IIA (green; IIA antibody) (**H, K**) in E16.5 femurs in WT (**G–I**) and S1P*^cko^* (**J–L**). Composite signals are shown in panels **I** and **L**. All images shown are for mature columnar proliferative chondrocytes in the WT and a corresponding region in S1P*^cko^*. Bar (all panels): 10 µm.

During synthesis, Col II is made as a procollagen of three identical pro-α-chains consisting of the amino (N) terminal propeptide, the THD domain, and the carboxy (C) terminal propeptide. The Col II protein incorporated into the mature cartilage is the THD domain with the N- and C-terminal propeptides removed. To understand the nature of the trapped Col II, we performed double-labeled immunofluorescence with the IIBN antibody [18], which detects the unique exon 1–3 peptide junction sequence specific to type IIB procollagen (pro-Col IIB) produced by chondrocytes (in contrast to the type IIA procollagen made by chondroprogenitor cells), in combination with anti-calnexin (Fig. 2A, B). In WT, signals from pro-Col IIB are undetectable presumably due to a rapid processing of the procollagen (Fig. 2A) [18]. In the composite picture for the WT (pro-Col IIB + calnexin; Fig. 2A) only signals from calnexin are seen showing the presence of the ER compartments. In contrast in S1P*^cko^*, significant signals from pro-Col IIB are detected that overlaps with signals from calnexin inside the cell (Fig. 2B).

When we repeated the analysis for pro-Col IIB in combination with IIF antibody (Fig. 2C–F), only the Col II THD is seen in the WT cartilage matrix indicating the presence of the mature processed Col II protein (Fig. 2C, E). However, hardly any Col II THD is detected in the S1P*^cko^* cartilage (Fig. 2D, F; also see Fig.1E). Signals from Col II THD is seen primarily trapped inside the cells (Fig. 2D) that overlaps with intracellular signals from pro-Col IIB (Fig. 2F). These observations indicate that in the mutant, the pro-Col IIB isoform of Col II is trapped in the ER. In order to analyze if this retention is specific to pro-Col IIB, we repeated the analysis using the IIA antibody specific to type IIA procollagen (Col IIA). In the WT (Fig. 2G–I), Col IIA is detected only outside the cell in the cartilage ECM where it forms an organized lattice network (in green, Fig. 2H). Importantly, an organized lattice network specific to Col IIA is also seen in S1P*^cko^* cartilage. The IIF antibody as seen before did not identify any Col II network in the mutant (compare Fig. 2I to 2L). These observations indicate that the absence of S1P activity affects only pro-Col IIB trafficking from the ER, and not Col IIA.

**Figure 3:**
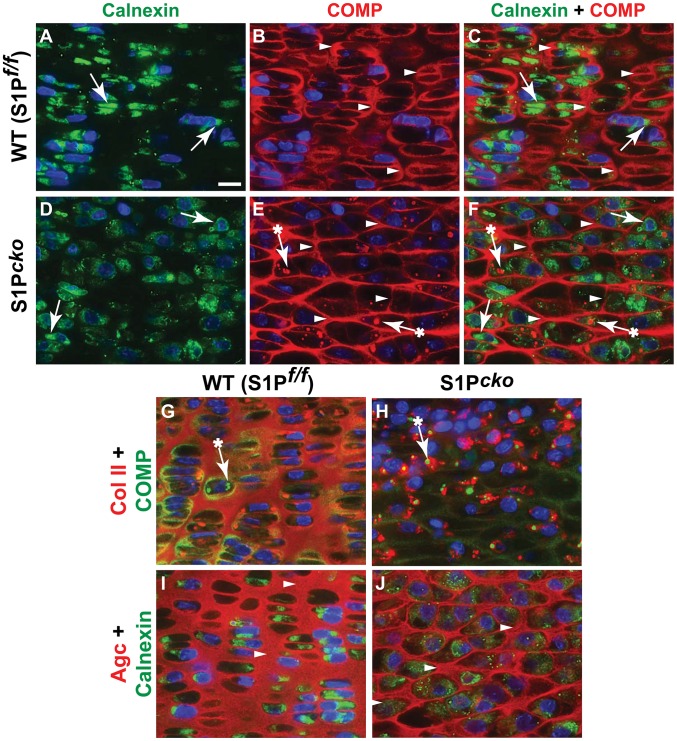
COMP and aggrecan are not intracellularly retained in S1P*^cko^* chondrocytes. Double-labeled immunofluorescence analyses for COMP and calnexin in E16.5 femurs in WT (A–C) and S1P*^cko^* (**D–F**). Individual signals from calnexin (**A, D**) are shown in green (white arrows) and those from COMP (**B, E**) are shown in red (white arrowheads), with composite signals in **C**, **F**. The relative distribution of Col II THD (red) and COMP (green) proteins for WT (**G**) and S1P*^cko^* (**H**) are shown as composites from double-labeled immunolabeling analyses in E16.5 femurs. Occasional entrapment of COMP is indicated by arrows marked with an asterisk. Composite signals showing the localization of aggrecan (Agc) (red) relative to calnexin (green) in E16.5 WT (**I**) or S1P*^cko^* (**J**) femurs. All images shown are for mature columnar proliferative chondrocytes in the WT and a corresponding region in S1P*^cko^*. Bar (all panels): 10 µm.

Next, we analyzed if trafficking of other major matrix proteins such as COMP and aggrecan is affected in S1P*^cko^* (Fig. 3). In WT chondrocytes, COMP (in red) is visible in the cartilage (white arrowheads, Fig. 3B, C) and its localization is distinct from calnexin (in green, white arrows, Fig. 3A,C) which surrounds the DAPI-stained blue nuclei. The distribution of COMP in the WT cartilage matrix is similar to that of Col II (compare Fig. 3B to Fig. 1B or 2C, E). Therefore when we labeled both Col II THD (red) and COMP (green) in the WT (Fig 3G), the staining patterns for Col II and COMP overlapped in the matrix; the cartilage is seen as yellowish-green in most areas with very little Col II or COMP detected intracellularly. Interestingly, the S1P*^cko^* cartilage also has a COMP-derived matrix (Fig. 3E, F). But COMP distribution does not resemble that seen in the WT cartilage and appears disordered and filamentous. In fact, COMP distribution in S1P*^cko^* cartilage approximates the distribution of Col IIA (compare Fig. 3E to 2K). This is probably due to the absence of the Col II THD domain in the S1P*^cko^* cartilage. Labeling S1P*^cko^* cartilage for both Col II (red) and COMP (green) (Fig. 3H) showed that COMP and Col II occupy distinct domains. While most of Col II is detected intracellularly, the majority of COMP is seen as a network in the cartilage. While some COMP is also detected trapped in the ER in S1P*^cko^* chondrocytes (white arrows with asterisk, Fig. 3E, H), these are usually very small aggregates and are usually surrounded by the larger Col II aggregates (Fig. 3H) suggesting retention due to a physical interaction with Col II (see DISCUSSION). Moreover, this retention of COMP is insignificant when compared to pro-Col IIB retention and has no consequence in the formation of a COMP matrix in the mutant cartilage. Co-localization studies with aggrecan (Agc) show that like COMP, S1P ablation does not affect its secretion (Fig. 3J). Thus our studies demonstrate that pro-Col IIB is the primary cartilage matrix protein trapped in the ER of S1P*^cko^* chondrocytes.

### Genome-wide expression profiling

The primary, well studied role of S1P is its transcription factor processing activity. Lack of transcription factor processing by S1P would affect both UPR and cholesterol/fatty acid homeostasis, which could be causal to S1P*^cko^* phenotypes. To understand the mechanisms behind this phenotype we profiled gene expression in mutant cartilage by microarray and contrasted it to WT cartilage to identify the biological processes disrupted in S1P*^cko^* chondrocytes. Statistically significant genes were identified by 2-way ANOVA analysis coupled with FDR of 5% (only 5% genes identified in the analysis could be false positive). Only genes that were 2-fold or more differentially regulated in the mutant were considered. Our primary aim was to understand the difference in gene expression in the chondroepiphyseal cartilage which at embryonic stages is the bulk of the cartilage in the skeletal elements. While S1P*^cko^* does not have any endochondral bone, it does have an enhanced cortical bone. Therefore entire long bones were also harvested and compared in this study. The Illumina MouseWG-6 v1.1 Expression BeadChip used in this study was chosen as it profiles more than 45,200 mouse transcripts including probes derived from the Mouse Exonic Evidence Based Oligonucleotide set and some less-characterized genes/probe sets derived from exemplar protein coding sequences from RIKEN FANTOM2.

**Figure 4:**
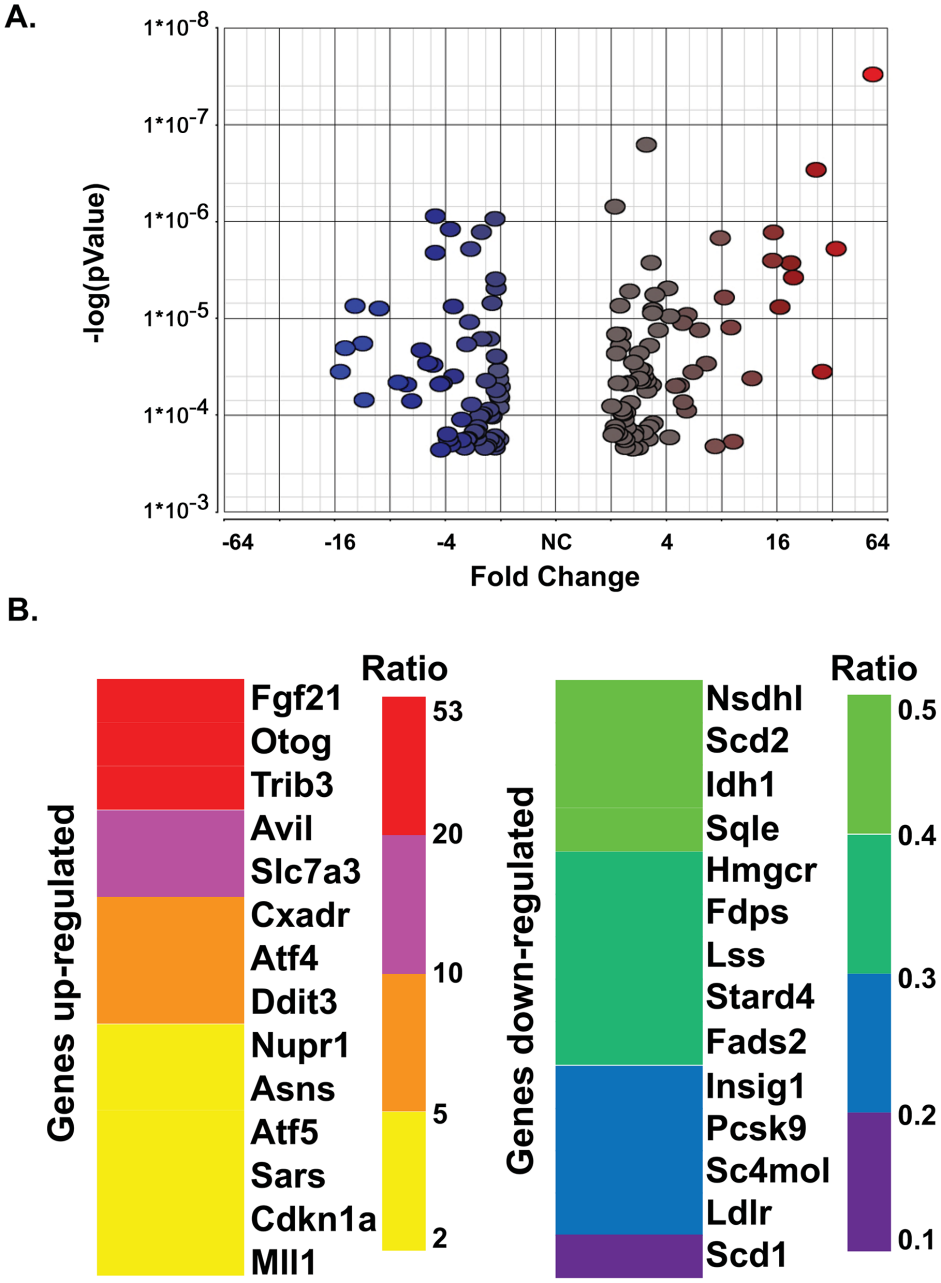
(**A**) A volcanic plot of the 145 differentially regulated genes in the S1P*^cko^* cartilage identified by 2-way ANOVA analysis. Only genes that show at least a 2-fold differential regulation with a *p*<0.05 (FDR ≤0.05) are shown, with the color red and its gradations identifying up-regulated genes and the color blue and its gradations identifying down-regulated genes. (**B**) Schematic representation of critical genes up- or down-regulated in S1P*^cko^* chondrocytes as compared to WT showing the range of differential expression in the mutant (un-abbreviated gene names are listed in Tables S3 and S4).

A principal components analysis (PCA) scatter plot representing only 40.8% of the whole genome information present in the microarray hybridization signals demonstrated that very little difference in gene expression is seen between the WT and S1P*^cko^* calvaria (Figure S1C). This suggested that skeletal structures arising from intramembranous ossification were likely not as affected by S1P ablation presumably because this developmental pathway does not need a cartilage intermediate. The PCA analysis also revealed that the largest difference in gene expression profile between WT and S1P*^cko^* lay in the cartilage. A 2-way ANOVA analysis coupled with a minimum of +2 to -2 differential fold regulation generated a stringent list of genes differentially regulated in S1P*^cko^* chondrocytes. Comparison of the gene expression profile between WT and S1P*^cko^* long bones showed similar data (not shown) presumably due to the presence of RNA from the cartilage, indicating that the difference lay primarily in the cartilage. Incorporation of calvaria data set in these analyses did not make any difference to the differentially expressed genes identified. Tables S3 and S4 list genes that are significantly differentially regulated (up- or down-regulated, respectively) in S1P*^cko^* chondrocytes. Figure 4A shows a volcano plot of the ANOVA analysis representing genes that are significantly differentially regulated (as per their *p* values) and exhibit fold changes of 2 or more. Figure 4B shows the range of differential regulation in S1P*^cko^* chondrocytes and the identity of some critical genes.

**Figure 5:**
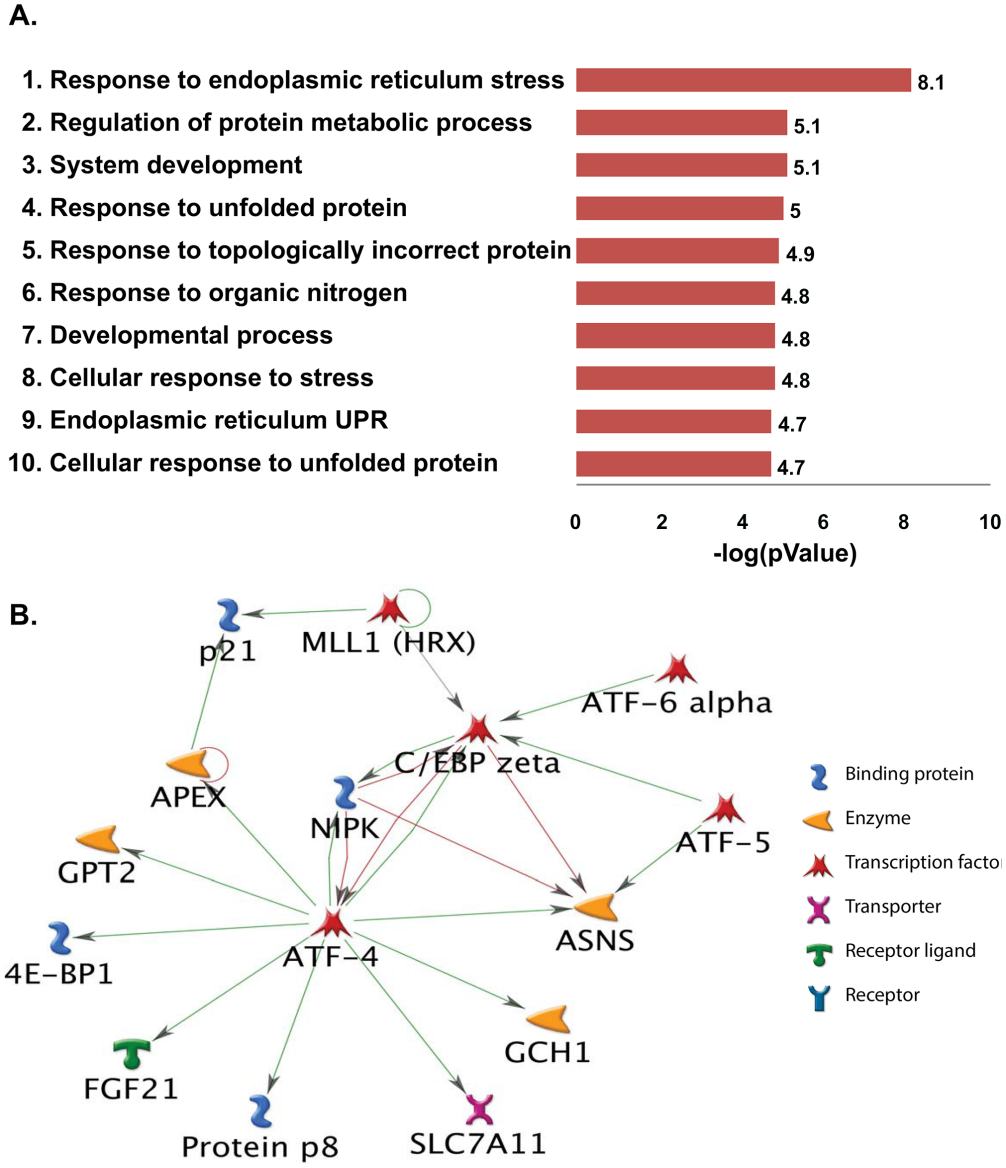
(**A**) A schematic representation of the top ten biological pathways up-regulated in S1P*^cko^* chondrocytes identified by GeneGo (MetaCore) based on their *p* value. The majority of up-regulated pathways belong to UPR in response to ER stress. (**B**) A molecular network generated from upregulated genes with the addition of ATF6α to the network. Green lines represent activation and red lines represent inhibition.

**Figure 6:**
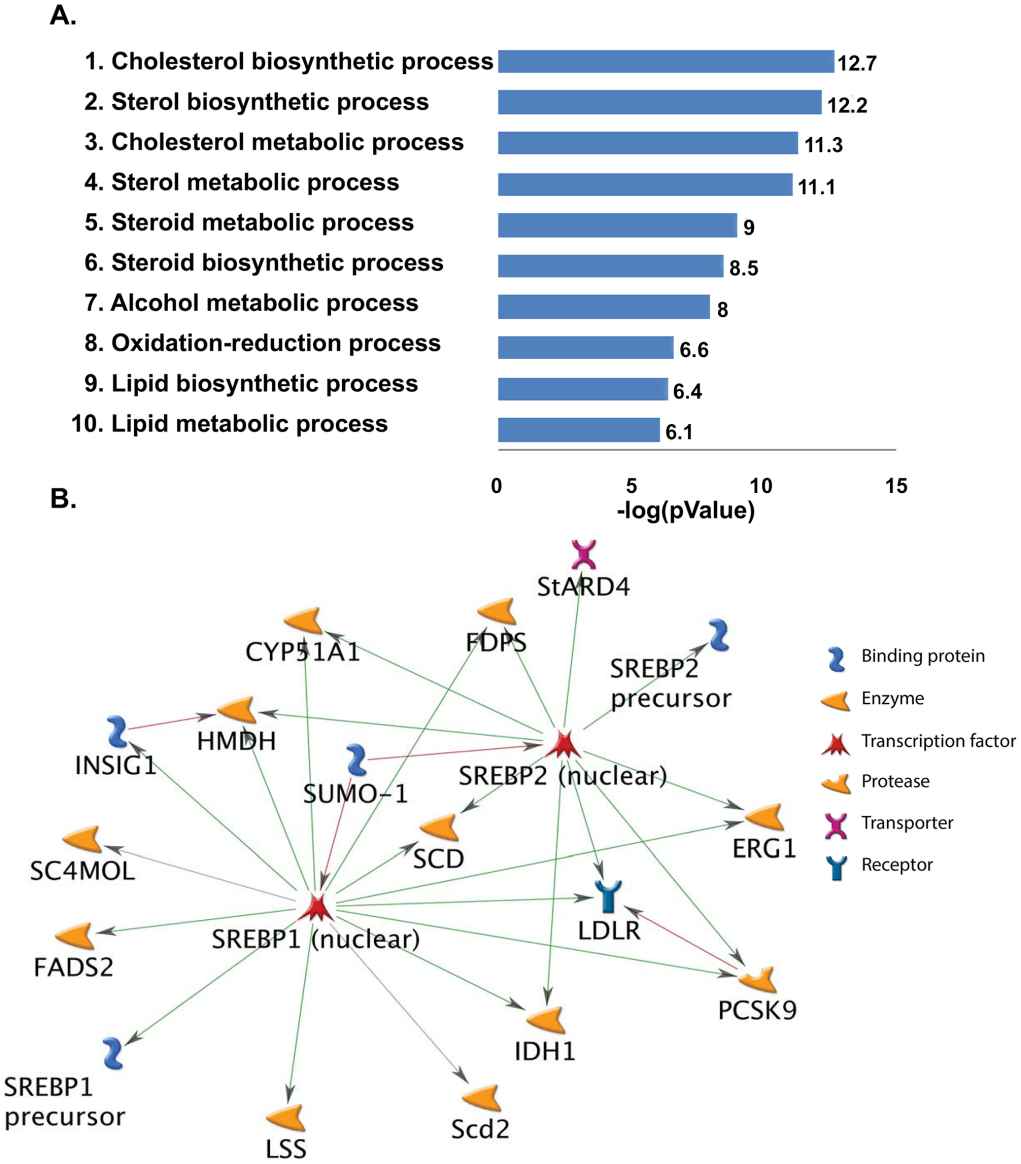
(**A**) A schematic representation of the top ten biological pathways down-regulated in S1P*^cko^* chondrocytes as identified by GeneGo based on their *p* value. The majority of down-regulated pathways identified belong to cholesterol and lipid biosynthetic pathways. (**B**) A molecular network generated from down-regulated genes with the addition of SREBPs to the network. Green lines represent activation and red lines represent inhibition.

Analyses of differentially expressed genes to identify functional biological pathways disrupted in the cartilage due to S1P ablation were done by GeneGo (MetaCore). Surprisingly, this analysis showed that the top 10 processes upregulated in S1P*^cko^* belonged primarily to UPR pathways (Fig.5A). This suggested that ER stress response pathways were unaffected in S1P*^cko^* cartilage. Furthermore, when a functional molecular network was created with these up-regulated genes with the addition of ATF6α to the network (Fig. 5B), a number of genes were found to be connected to ATF6α, directly or indirectly, and also to each other, acting in concert to respond to ER stress due to pro-Col IIB entrapment in S1P*^cko^* chondrocytes. This suggested that ATF6 activity was unlikely to be disrupted in S1P*^cko^*. When the significantly differentially down-regulated genes were analyzed similarly by GeneGo, the top 10 down-regulated processes belonged primarily to cholesterol and lipid biosynthetic pathways (Fig. 6A). As with the up-regulated genes, when a molecular network was created with the down-regulated genes with the addition of SREBPs to the network, a large number of these genes were found to be interconnected with SREBP-1 and SREBP-2 (Fig. 6B) indicating that this is not an isolated event but are molecular events acting in concert to down-regulate cholesterol and lipid biosynthesis.

### Intact unfolded protein response in S1P^cko^ chondrocytes

The UPR is modulated by the action of three distinct pathways via three distinct ER transmembrane sensors, IRE1 (inositol-requiring transmembrane kinase and endonuclease 1), PERK (protein kinase-like ER kinase), and ATF6 [26]–[28]. Activation of IRE1 results in the endonucleolytic cleavage of XBP-1 mRNA to allow for frame-shift translation and expression of the active XBP-1 transcription factor, which increases the expression of UPR-responsive genes. Activation of PERK pathway leads to phosphorylation of elongation initiation factor 2α thereby inactivating it, which attenuates translation thus decreasing the protein burden of the cell. Furthermore, this diminished translation allows for the selective increased translation of ATF4 which alone or in combination with XBP-1 allows for expression of UPR-responsive genes. Release of ATF6 from the ER by the combined action of S1P and site-2 protease (S2P) and its translocation to the nucleus, in cooperation with NF-Y or XBP-1, up-regulates transcription of UPR-responsive genes.

An indication that the PERK pathway is normal in S1P*^cko^* is seen by the up-regulation of ATF4 identified in the microarray analysis (Fig. 4B, 5B). This is further confirmed by the increased expression of ATF4-controlled genes such as DNA damage inducible transcript 3 (Ddit3, also known as C/EBP zeta/CHOP/CHOP10; Fig. 4B, 5B) [29]. Ddit3 is known to be involved in a variety of ER stress response pathways [30], [31]. It is also known to induce apoptosis in response to ER stress [32]. This up-regulation is in consensus with the drastic increase in apoptosis seen in S1P*^cko^* cartilage [15]. ATF4 also up-regulates genes involved in amino acid metabolism that may protect against oxidative stress by promoting glutathione synthesis. The microarray analysis also showed increased expression of two well known markers of this response, the amino acid transporter protein solute carrier family 7 (Slc7a3) and the amino acid biosynthetic enzyme asparagine synthetase (Asns) (Fig. 4B and 5B) [33], [34]. Up-regulation of several aminoacyl tRNA synthetases such as seryl amino-acyl tRNA synthetase (Sars) and alanyl tRNA synthetase (Aars) is also seen (Fig. 4B). Advanced ER stress response is also indicated by another target of ATF4-CHOP, namely tribbles 3 (Trib3/Nipk) (Fig. 4B, 5B), an ER stress inducible gene, which also functions by down-regulating its own induction to attenuate CHOP and ATF4 mediated transcriptional events [35]. Induction of the cell cycle inhibitor p21 (Cdkn1a) (Fig. 4B, 5B), a negative regulator of apoptosis [36] could be the cell's attempt to antagonize increased apoptosis.

**Figure 7:**
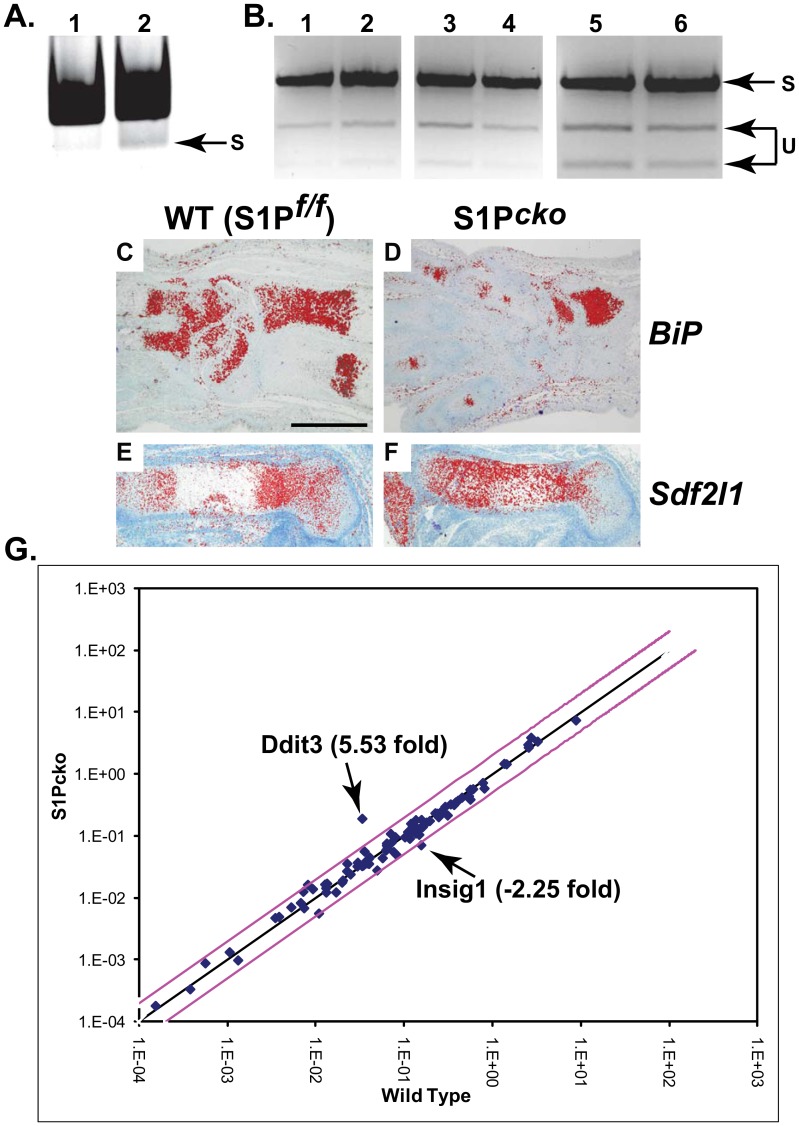
Intact ER stress response in S1P*^cko^* chondrocytes. (**A**) RNA from the chondroepiphyseal cartilage of E16.5 WT (lane 1) and S1P*^cko^* (lane 2) used in microarray analysis were converted to cDNA and amplified by XBP-1 PCR primers to identify spliced (s) XBP-1 mRNA in a 4.8% polyacrylamide gel. (**B**) XBP-1 was amplified by PCR using XBP-1 PCR primers 3S and 12AS with cDNA derived from E16.5 WT (lanes 1, 3, 5) and S1P*^cko^* (lanes 2, 4, 6) epiphyseal cartilage RNA and the PCR product restriction digested with *Pst*I which selectively cuts the un-spliced (u) XBP-1 mRNA, and the resulting products visualized in a 2% agarose gel. In lanes 1 and 2, the cDNA used are from the same embryos used in (**A**) and for genome-wide expression profiling. Each lane in lanes 3–6 show analyses from RNA pooled from the chondroepiphysis of two different embryos. Thus a total of five WT and five S1P*^cko^* embryos were analyzed. The inverse of the gels are shown in both (**A**) and (**B**) to enhance visualization of spliced XBP-1 mRNA. (**C–F**) Expression signaling for two ATF6-driven genes, BiP and Sdf2l1, as seen by in situ hybridization analyses in WT and S1P*^cko^* cartilage. BiP expression is seen in the ulna, carpal, and metacarpal regions in E16.5 WT (**C**) and S1P*^cko^* (**D**) forelimbs. Sdf2l1 expression is seen in the femur of E15.5 WT (**E**) and S1P*^cko^* (**F**). Bar: 10 µm. (**G**) A scatter plot generated from quantitative real-time PCR analysis in the murine UPR RT^2^ Profiler PCR Array system comparing the relative expression of 84 genes between WT and S1P*^cko^* chondrocytes. A log transformation plot is shown in which the relative gene expression level of each gene (2^-ΔCt^) in WT is plotted against the corresponding value in S1P*^cko^* to indicate fold changes (2^-ΔΔCt^). The black line indicates no fold change (fold change of 1). The pink lines indicate a fold change of 2 (gene expression threshold). All genes within these two lines are considered to be similar in expression to WT. Only Ddit3 or Insig-1 were significantly differentially expressed among the 84 genes profiled. A total of four WT and four S1P*^cko^* embryos were profiled.

To analyze events directed by the IRE1 pathway we analyzed XBP-1 splicing in S1P*^cko^* chondrocytes (Fig. 7A, B). Using XBP-1 primers whose sequences are common to both the spliced (s) and un-spliced (u) XBP-1 mRNA [21], [25] we checked the ability of the primers to generate the smaller, spliced XBP-1 mRNA product in PCR reactions using cDNA made from WT and S1P*^cko^* RNA used in the genome-wide expression profiling (Fig. 7A). Both WT and S1P*^cko^* cDNA showed the ability to generate the s-XBP-1 mRNA. We also confirmed XBP-1 splicing by coupling restriction digestion with PCR by using primers 3S and 12AS [22] on cDNA derived from the chondroepiphyseal cartilage RNA of more than one E16.5 WT and S1P*^cko^* embryo (Fig. 7B). Again, both WT and S1P*^cko^* showed the ability to splice XBP-1 mRNA suggesting an intact IRE-1 directed UPR system in the mutant chondrocytes.

Based on microarray analysis, we did not observe any down-regulation of any known ATF6-responsive genes during ER stress response. Genes whose expressions are driven by ATF6 such as glucose related protein 78 (Grp78/BiP/Hspa5), heat shock protein 90b1 (Hsp90b1/Grp94), derlin-3, and stromal cell-derived factor 2-like 1 (Sdf2l1), and are significantly down-regulated during ER stress induction in *ATF6−/−* cell lines [37] were not identified as differentially regulated in S1P*^cko^* cartilage. To confirm this observation we performed in situ hybridization analysis for Sdf2l1 and BiP. Significant expression of these genes was detected in S1P*^cko^* cartilage (Fig. 7D, F). In order to validate the microarray results and to get a definitive, global answer about ER stress response we performed qPCR analysis by using the murine Unfolded Protein Response RT^2^ Profiler PCR Array system that profiles the expression of 84 genes (Table S2) involved in recognizing and responding to misfolded protein accumulation in the ER (Fig. 7G). For this assay we used fresh RNA isolated from the chondroepiphysis of four WT and four S1P*^cko^* E16.5 embryos allowing for biological duplicates. Only two significant deviations from WT cartilage was observed, namely Ddit3 and Insig-1, which were also identified from microarray studies (Fig. 4B). The rest of the genes profiled including genes such as BiP (Hspa5), Herp, and Edem1, whose expressions were down-regulated on ER stress in *ATF6−/−* cell lines, did not show any significant change in the mutant. These observations confirmed normal UPR responses in S1P*^cko^*.

### Down-regulation of SREBPs-responsive genes in S1P^cko^ cartilage

**Figure 8:**
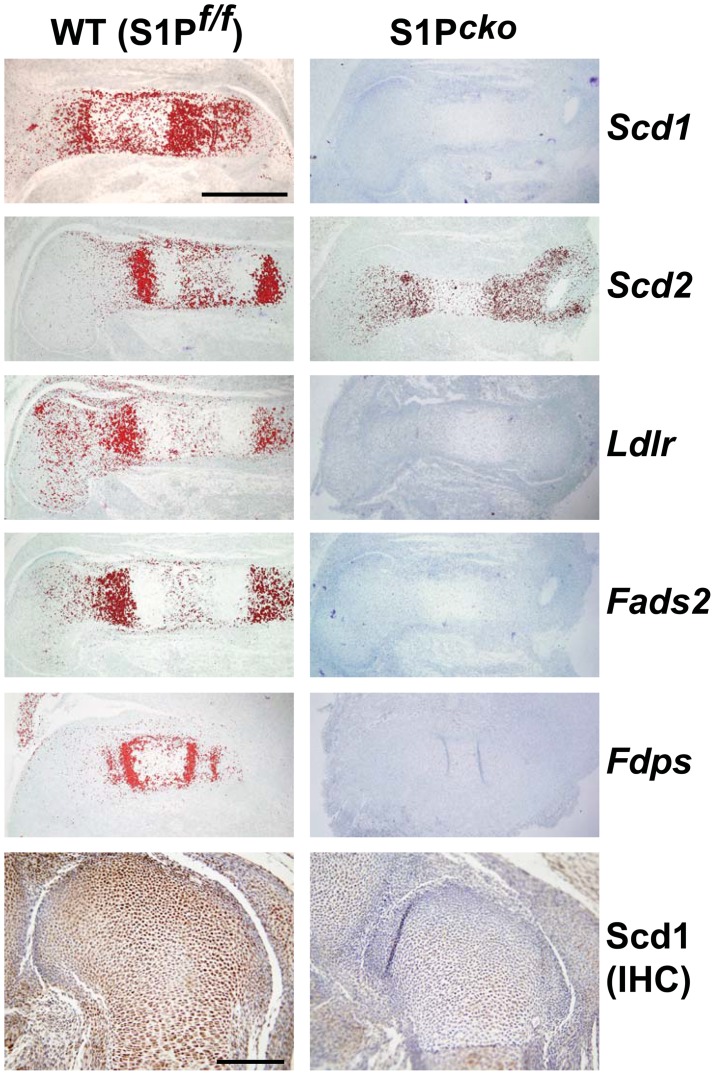
Expression analyses of some key genes involved in cholesterol and fatty acid biosynthesis in S1P*^cko^* cartilage by in situ hybridization. Notice the drastic reduction in the expression of Scd1, Scd2, Ldlr, Fads2, and Fdps genes at the RNA level, shown by in situ hybridization in S1P*^cko^* humerus and by immunohistochemistry (IHC) for Scd1 protein in S1P*^cko^* femur. All analyses are shown at E15.5 except for Fdps which is shown at E14.5. Bars: (in situ hybridization analyses): 500 µm; (IHC): 200 µm.

Figure 6 suggested that genes/pathways regulated by SREBPs involved in cholesterol and fatty acid biosynthetic processes are primarily down-regulated in S1P*^cko^* cartilage. In order to confirm this observation we performed qPCR analyses of a number of genes identified as down-regulated (see Table S4). A number of these are known to be direct targets of S1P/SREBP regulatory pathway, i.e. genes reported to have a sterol regulatory element (SRE) motif in their promoter and requiring SREBPs as transcription factors for their synthesis [38] (see Fig. 4B, 6B). As with the qPCR-based global UPR analyses above, we used fresh RNA pooled from the chondroepiphysis of five E16.5 WT or five S1P*^cko^* embryos. A number of genes identified as down-regulated by microarray analysis such as Fgfr1op2, Sparc, and Loricrin did not show down-regulation in mutant chondrocytes on qPCR analysis (not shown). Along with Prg4 (which is not reported to use SREBPs as transcription factors for its expression; see DISCUSSION), qPCR analyses confirmed that genes such as Scd1, Sc4mol, Fads2, Scd2, Stard4, Ldlr, Insig-1, and Fdps, which are direct targets of SREBPs and involved in the cholesterol/lipid biosynthetic pathways, are down-regulated in S1P*^cko^* cartilage (Table 2) mirroring that seen in the microarray. Especially significant among these genes was Scd1 which exhibited the largest down-regulation. We further validated these observations by in situ hybridization analysis demonstrating the down-regulation of Scd1, Scd2, Ldlr, Fads2, and Fdps genes at the RNA level in the humerus of S1P*^cko^* mice (Fig. 8). Immunohistochemistry also confirmed the down-regulation of the Scd1 protein in the distal femoral cartilage in S1P*^cko^* mice (Fig. 8). These analyses confirmed the significant down-regulation of cholesterol and fatty acid biosynthetic pathways in the mutant and suggested that this down-regulation may be causal to the abnormal cartilage ECM and lack of endochondral bone development.

**Table 2:** Quantitative real-time PCR analyses of genes down-regulated in S1P*^cko^* chondrocytes when compared to WT littermates.

Gene	Wild Type	S1P*^cko^*
Scd1	1.00	0.27
Sc4mol	1.00	0.41
Fads2	1.00	0.54
Scd2	1.00	0.57
Stard4	1.00	0.58
Ldlr	1.00	0.59
Insig-1	1.00	0.63
Fdps	1.00	0.65
Hmgcs	1.00	0.86
Hmgcr	1.00	0.91

RNA was harvested and pooled from the chondroepiphyseal cartilage of five E16.5 S1P*^cko^* or five E16.5 WT embryos.

**Figure 9:**
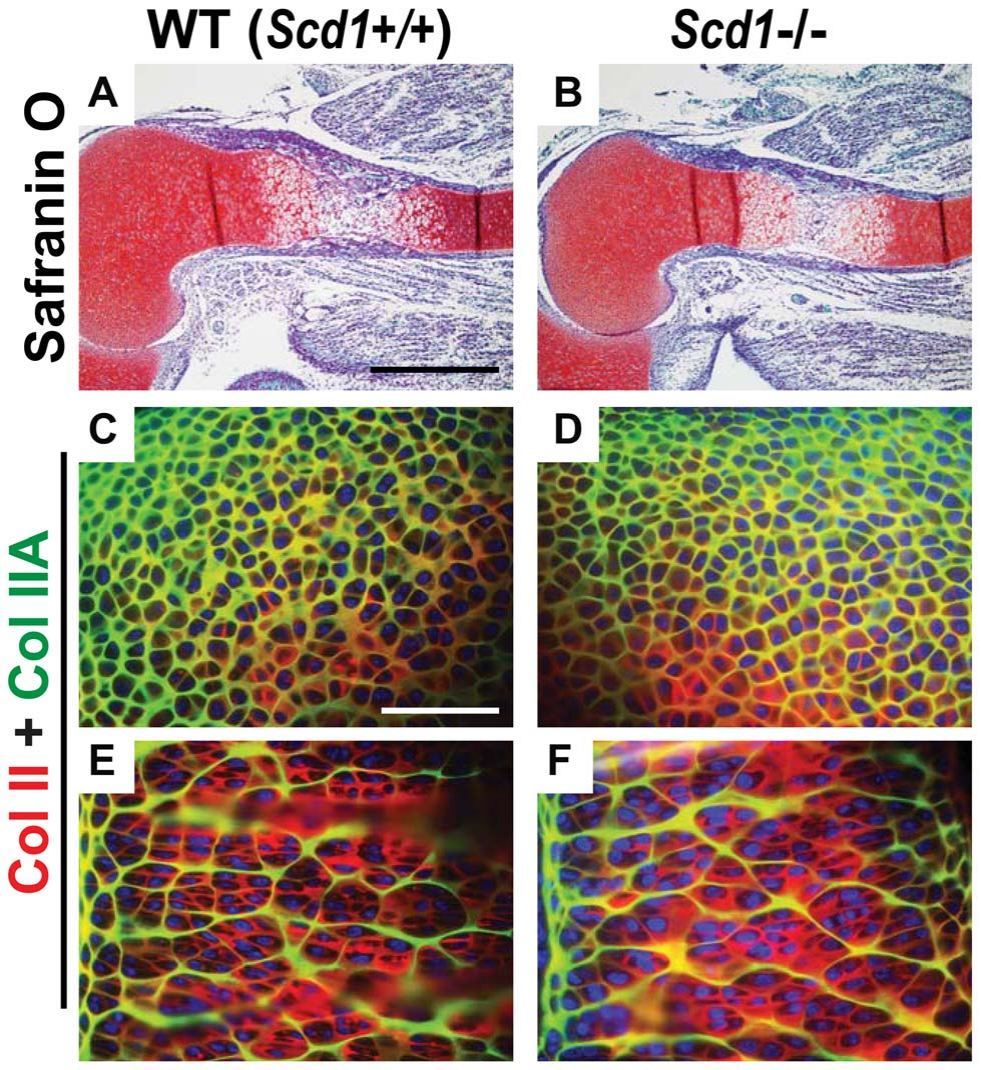
Normal endochondral bone development and Col II deposition in *Scd1*−/− mice. (**A, B**) Sections of E15.5 humerus were stained with Safranin O, Fast green, and hematoxylin showing normal onset of endochondral bone development in *Scd1*−/− mice. (**C–F**) Double-labeled immunofluorescence analyzing Col II deposition in *Scd1*−/− mice using IIF and IIA antibodies. Colors represent antibody localizations as follows: green, Col IIA (IIA antibody); red, Col II THD (IIF antibody); yellow, colocalization of both antibodies; blue, DAPI-stained nuclei. Panels **C** and **D** show the matrix around early immature chondrocytes in the resting zone; panels **E** and **F** show the matrix around mature columnar chondrocytes in the proliferative zone. Bar: (A, B): 500 µm; (C–F): 50 µm.

To follow this observation further, we studied Col II retention and endochondral bone formation in Scd1 knockout (*Scd1−/−*) mice. Among genes with a direct molecular link to SREBPs, the Scd1 gene was maximally down-regulated and thus could be considered a good candidate to induce the S1P*^cko^* phenotypes. Scd1 enzyme catalyzes the Δ9-desaturation of saturated fatty acids such as palmitic acid and stearic acid to their corresponding monounsaturated fatty acids (MUFA), palmitoleate and oleate, respectively. As membranes are composed of lipids, we hypothesized that retention of matrix proteins in the ER and the resulting ER stress could be due to ER membrane lipid changes. Changes in MUFA composition of the ER membrane and the resulting changes in membrane fluidity could result in Col II retention in the ER. To understand whether the phenotype in S1P*^cko^* mice is driven by lack of Scd1 activity, we analyzed endochondral bone formation and Col II deposition in *Scd1−/−* mice. *Scd1*−/− mice not only suffer from the down-regulation of Scd1 activity but also demonstrated down-regulation in lipid biosynthesis [22], [39], [40], a situation that could be considered analogous to the down-regulation of cholesterol and lipid homeostasis seen in S1P*^cko^* chondrocytes. Interestingly however, onset of endochondral bone formation and Col II deposition in *Scd1*−/− mice were similar to that seen in wild type (*Scd1+/+*) mice (Fig. 9).

## Discussion

The disruption of Col II secretion is central to S1P ablations [15], [41]. In this study we demonstrated that S1P ablation in cartilage results in the specific retention of the pro-Col IIB isoform in the chondrocyte ER. As the Col2-Cre system initiates deletion of S1P very early at E11.5-E12 [42], chondroprogenitor cells which synthesize Col IIA also suffer from S1P ablation. However this has no effect on Col IIA secretion, a difference probably mediated by the presence of exon 2 coded sequences in the N-propeptide of Col IIA. Thus, pro-Col IIB is the major cartilage protein retained in mutant chondrocytes which prevents the formation of a Col II matrix in S1P*^cko^* cartilage. This indicates that S1P ablation disrupts pro-Col IIB trafficking from the ER and that a S1P-derived activity is essential for pro-Col IIB trafficking from the ER to the cartilage. The minor retention of COMP is presumably due to interactions between small amounts of COMP with the large pro-Col IIB aggregates in the ER. This phenomenon is not unique to S1P*^cko^* and has been observed in pseudoachondroplasia (PSACH) chondrocytes. PSACH chondrocytes express mutant COMP which causes its retention in the ER. Interactions between the trapped mutant COMP and Col II results in matrix formation inside the ER [43] with Col II aggregates surrounded by a COMP matrix. These observations indicate that the minor retention of COMP seen in S1P*^cko^* cartilage is incidental to pro-Col IIB retention. The dynamic association between Col II and COMP in the cartilage is also evidenced by the similarity and overlap of their distribution in WT cartilage and the absence of a similar COMP organization in S1P*^cko^* cartilage due to the lack of a pro-Col IIB-derived Col II matrix.

In order to identify the molecular pathways affected by S1P ablation that disrupted pro-Col IIB trafficking, we performed genome-wide expression analysis in the S1P*^cko^* model. Genome-wide expression profiling on RNA isolated from the chondroepiphyseal cartilage of E16.5 S1P*^cko^* and WT littermates followed by 2-way ANOVA and gene ontology enrichment analysis demonstrated that only the SREBPs-regulated pathways are affected in S1P*^cko^* chondrocytes. The Prg4 (lubricin) gene which has no reported association with S1P or SREBPs or any SRE motif in its promoter was also found to be down-regulated. However, knockout mouse models for Prg4 [44] do not show any defects in endochondral bone formation or Col II trafficking to the ECM. Therefore, it seems possible that Prg4 down-regulation in S1P*^cko^* cartilage is a secondary effect due to poor cartilage matrix development in these mice or due to abnormal chondrocyte differentiation.

As chondrocytes secrete large amounts of various cartilage matrix proteins to assemble the specialized cartilage ECM, a functional UPR would be indispensable to this function. Thus we had hypothesized that S1P ablation would prevent an effective UPR response to ER stress which would cause intracellular Col II accumulation [15]. However, UPR response pathways are unaffected in S1P*^cko^* chondrocytes. The activity of ATF6 is unchanged on S1P ablation and there is a lack of differential expression of ATF6-responsive genes such as BiP and Sdf2l1. This can be explained by reasoning that the requirement of S1P for ATF6 processing is not absolute, which is also noted in other systems. ER stress response was reported to be normal in zebrafish *gonzo* phenotype where expression of BiP was shown as unaltered in absence of S1P [6]. The S1P-lacking SRD-12B cells demonstrated only a partial ability to prevent ATF6 processing to its active form on induction of ER stress; only S2P ablation allowed for a complete lack of ATF6 processing [45]. Thus, even moderate amounts of active ATF6 in concert with other nuclear factors such as NF-Y [30] and/or XBP-1 [46] would have sufficient transcriptional induction properties to induce UPR. Thus, in this study it was not possible to address the importance of UPR to cartilage ECM development. However, viable and fertile ATF6α knockout mice were reported [46] suggesting that ATF6-directed ER stress responses are not necessary for normal Col II deposition or endochondral bone development in mice. Notably, these studies indicate that the apoptosis seen in S1P*^cko^* cartilage is due to the trapped Col II in the ER and induction of UPR, rather than an inability to respond to ER stress.

Other ER stress transducers such as OASIS and its related family members BBF2H7 and CREBH also appear to be unaffected. OASIS has a specific expression pattern and is primarily induced in bone tissues and the central nervous system, but no expression in chondrocytes has been reported [47]; likewise CREBH is liver-specific [5]. Thus OASIS and CREBH and their dependent pathways are not relevant in S1P*^cko^*. But BBF2H7 differs from OASIS in its strong expression in the proliferating zone of the cartilage [48]; it has also been reported to need S1P for its processing [49] and would be expected to be inactive in S1P*^cko^* chondrocytes. *Bbf2h7-/-* mice show severe chondrodysplasia and the retention of Col II in the ER similar to that seen in S1P*^cko^* mice [48]. BBF2H7 is required for the expression of Sec23a protein, a component of COPII vesicles which transports secretory proteins from ER to the matrix. However, Sec23a is unaffected in S1P*^cko^* chondrocytes as it was not identified as differentially down-regulated in the microarray analysis and was further validated by qPCR analysis (Figure S2); nor were its protein levels affected (not shown). Therefore, in S1P*^cko^* chondrocytes, ER stress transducers and their activities have remained largely unaffected.

However the requirement for S1P is absolute for the SREBPs. SRD-12B (*S1P−/−*) cells, which only partially prevent ATF6 processing, are able to impose a complete block of SREBPs processing [45], [50]. This would explain the specific down-regulation of SREBP-driven pathways in S1P*^cko^* chondrocytes. However, this down-regulation does not appear to be responsible for the mutant phenotypes seen on S1P ablation in chondrocytes. We had surmised that potential changes in ER membrane composition and fluidity accompanying the down-regulation of sterol and lipid pathways may be responsible for Col II retention seen in the ER. If this was true then these ER membrane changes would also be present in the chondrocyte ER of *Scd1−/−* mice that would lead to abnormal cartilage and endochondral bone development. However, we observed no intracellular Col II retention or lack of endochondral bone development in *Scd1−/−* mice. It is also striking that mice lacking Ldlr [51], [52] or Fads2 (Delta-6 desaturase) [53], genes that are also down-regulated in S1P*^cko^* chondrocytes, show no disruption in endochondral bone development as that seen in S1P*^cko^*. Furthermore, neither are these mutant phenotypes seen in mouse models with compound or multiple disruptions in lipid homeostasis such as *Ldlr−/−;Lcat−/−* mice [51].

These observations may be explained by the possibility to separate cartilage developmental pathways from lipid pathways. In the zebrafish *gonzo* phenotype, S1P ablation results in both cartilage and lipid phenotypes. But ablation of SREBP cleavage-activating protein (SCAP) results only in lipid phenotypes but no cartilage phenotype [6]. This indicates that lipid and cartilage defects are caused by different mechanisms. However, it has been reported that the inhibition of cholesterol biosynthesis through the administration of a chemical inhibitor AY 9944 in mice suppressed longitudinal bone growth via suppression of chondrocyte proliferation and hypertrophy [54]. These contrasting observations make it difficult to discern the exact mechanistic input of sterol and lipid homeostasis to cartilage development. It is possible that cholesterol/lipid down-regulation and ER entrapment of Col II are independent phenotypes from two separate functions of S1P, but those functions relating to Col II trafficking remain to be identified.

**Figure 10:**
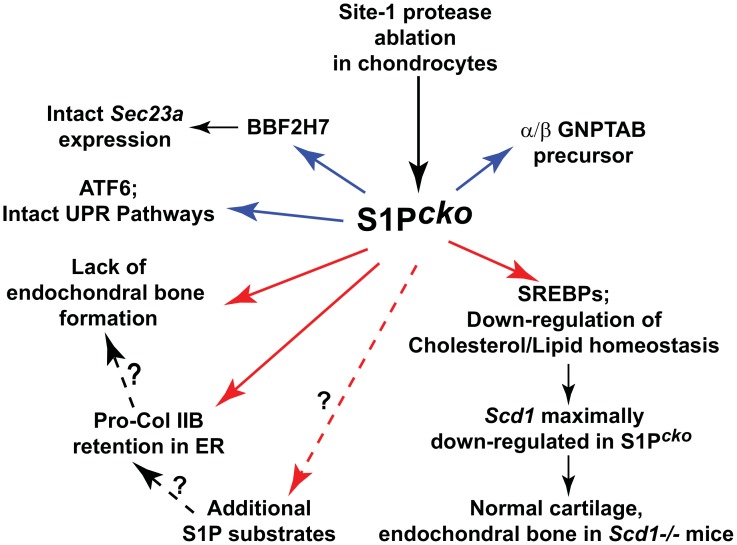
A schematic summarizing the results from this study and possible additional roles for S1P in cartilage development. S1P is historically known for processing the transcription factors ATF6 and SREBPs. Two relatively recently discovered activities include the processing of BBF2H7 and the α/β subunit precursor of N-acetylglucosamine-1-phosphotransferase complex (GNPTAB). Of these four known activities, only SREBPs activities relating to cholesterol and fatty acid homeostasis are down-regulated in S1P*^cko^* chondrocytes, with maximal down-regulation of Scd1. However this does not appear to be responsible for the S1P*^cko^* mutant phenotypes as pro-Col IIB retention in chondrocytes or lack of endochondral bone development is not seen in *Scd1−/−* mice. The retention of pro-Col IIB in the ER could be due to changes in ER membrane lipid composition, though unlikely as pro-Col IIB retention is not seen in *Scd1−/−* mice. Processing of additional, yet unidentified, S1P substrates in chondrocytes likely modulate pro-Col IIB trafficking from the ER. Lack of endochondral bone formation is probably due to the abnormal cartilage matrix devoid of Col II, or due to unidentified S1P-regulated processes. In the figure, blue arrows indicate activities that are normal in S1P*^cko^* chondrocytes; red arrows indicate activities that are down-regulated or abnormal in S1P*^cko^*. Dashed arrows with a question mark indicate the possibility of these mechanisms directed by novel S1P substrates, but nothing more is known as yet.

Figure 10 summarizes our current findings. In this study we investigated all current known functions of S1P: induction of ER stress response, induction of cholesterol and fatty acid biosynthesis, processing of BBF2H7 to effect Sec23a expression, and the processing of the α/β subunit precursor of N-acetylglucosamine-1-phosphotransferase complex (GNPTAB). Recently it was demonstrated that GNPTAB, involved in the addition of mannose 6-phosphate residues on lysosomal enzymes, is a direct S1P substrate [55]. In our preliminary experiments, there is at least equivalent GNPTAB α/β precursor cleavage activity in S1P*^cko^* chondrocytes as in chondrocytes from its wild type littermates (not shown). This indicates that this activity is not lost in S1P*^cko^* chondrocytes and therefore not causal to the mutant phenotypes. Besides, mucolipidosis II (*Gnptab^c.3082insC^*) mice (which lack GNPTAB) do not exhibit lack of endochondral bone formation or Col II entrapment in the ER [55]. Our studies thus indicate that activities associated with current known functions of S1P cannot provide for a direct molecular explanation of S1P*^cko^* phenotypes, especially the entrapment of pro-Col IIB in the ER. We therefore propose that S1P has additional, novel substrates in chondrocytes (schematized in Fig. 10), likely non-transcription factor-related, that is vital to normal cartilage development; at least one of these substrates is specific and indispensable for pro-Col IIB processing and/or trafficking from the ER. The identification of this novel S1P substrate will not only allow for a molecular understanding of S1P*^cko^* phenotypes, but will also allow for an unraveling of the molecular mechanism involved in pro-Col IIB trafficking, a phenomenon that still remains poorly understood. Our studies thus indicate an increased breadth of S1P functions in chondrocytes mandatory for normal cartilage development.

## Supporting Information

Figure S1 (**A**) **A diagrammatic representation of the WT embryonic growth plate.** All immunohistochemical analyses are shown for the columnar proliferative regions above the hypertrophic cells and for a corresponding region in S1P*^cko^*. The chondroosseous junction is the approximate region where the tips of long bones were broken off and harvested for cartilage analysis by microarray. S1P*^cko^* lacks endochondral bone and the diaphysis of the bone is cartilaginous. (**B**) A diagrammatic representation of the cartilage tissue harvested for microarray analysis. For analysis of WT and S1P*^cko^* cartilage by microarray, the tips of the long bones (shown demarcated by the dashed line and denoted as epiphyseal cartilage) were broken off and stored in Trizol. When entire long bones were harvested for analysis, the bones were harvested intact and the epiphyseal cartilage portions were not separated from the bone. (**C**) A schematic representation of the differences between WT (rectangles) or S1P*^cko^* (triangles) RNA using a principal component analysis scatter plot based on microarray hybridization signals. RNA was analyzed from cartilage (green), entire long bones (red), and calvaria (blue). Differential gene expression between WT and S1P*^cko^* calvaria (blue) is minimal (located very close to each other). Differential gene expression between WT and S1P*^cko^* cartilage (green) is maximum (located farthest from each other), while those between WT and S1P*^cko^* long bones (red) is intermediate to cartilage and calvaria.
(EPS)

Figure S2 **Expression levels for Sec23a are unaffected in S1P** ***^cko^*** **mice.** Each WT or S1P*^cko^* (CKO) analysis used RNA pooled from the chondroepiphyseal cartilage of two embryos. Thus a total of four WT and four S1P*^cko^* embryos were analyzed. Q-PCR analysis was done using the 2^-ΔΔCt^ method where the WT values were set to one.
(EPS)

Table S1 **SYBR Green qPCR primer sets for the murine genes listed were taken from the MGH/Harvard Medical School primer bank (** http://pga.mgh.harvard.edu/primerbank/ **).**
(DOCX)

Table S2 **A complete list of 84 genes that were profiled by qPCR using the murine Unfolded Protein Response RT^2^ Profiler PCR Array system, shown grouped according to their functions.**
(DOCX)

Table S3 **A partial list of genes significantly up-regulated in S1P** ***^cko^*** **chondrocytes identified from microarray analysis.** Shown are top 20 genes that are differentially up-regulated when compared to WT, and some selected genes. RNAs with no corresponding gene names are not included in this list.
(DOCX)

Table S4 **A partial list of genes significantly down-regulated in S1P** ***^cko^*** **chondrocytes identified from microarray analysis.** Shown are top 20 genes that are differentially down-regulated when compared to WT, and some selected genes that belong to fatty acid and cholesterol biosynthesis pathways. RNAs with no corresponding gene names are not included in this list.
(DOCX)
