# Supplementary material for: Cartilage-Specific Ablation of Site-1 Protease in Mice Results in the Endoplasmic Reticulum Entrapment of Type IIB Procollagen and Down-Regulation of Cholesterol and Lipid Homeostasis
Source: PLoS One. 2014 Aug 22;9(8):e105674. doi: 10.1371/journal.pone.0105674 (PMC4141819; doi:10.1371/journal.pone.0105674)
Supplement: Table S1 — SYBR Green qPCR primer sets for the murine genes listed were taken from the MGH/Harvard Medical School primer bank ( http://pga.mgh.harvard.edu/primerbank/ ). (DOCX) [file pone.0105674.s003.docx]

**Table S1**

SYBR Green qPCR primer sets for the murine genes listed were taken from the MGH/Harvard Medical School primer bank (<http://pga.mgh.harvard.edu/primerbank/>).

| **Gene** | **Forward Primer** | **Reverse Primer** |
| --- | --- | --- |
| Gapdh | 5’-AGGTCGGTGTGAACGGATTTG-3’ | 5’-TGTAGACCATGTAGTTGAGGTCA-3’ |
| Fads2 | 5’-TCATCGGACACTATTCGGGAG-3’ | 5’-GGGCCAGCTCACCAATCAG-3’ |
| Sc4mol | 5’-AAACAAAAGTGTTGGCGTGTTC-3’ | 5’-AAGCATTCTTAAAGGGCTCCTG-3’ |
| Stard4 | 5’-AGCCCGTGGTCACAGATTG-3’ | 5’-GCAACTCGCCACTCATCTTC-3’ |
| Lor | 5’-CTCCTGTGGGTTGTGGAAAGA-3’ | 5’-TGGAACCACCTCCATAGGAAC-3’ |
| Prg4 | 5’-GAAAATACTTCCCGTCTGCTTGT-3’ | 5’-ACTCCATGTAGTGCTGACAGTTA-3’ |
| Fgfr1op2 | 5’-GAAGGCACTTGCTGACGCTAA-3’ | 5’-GCTTCCACTCGCTTGCTGA-3’ |
