# Supplementary material for: Cartilage-Specific Ablation of Site-1 Protease in Mice Results in the Endoplasmic Reticulum Entrapment of Type IIB Procollagen and Down-Regulation of Cholesterol and Lipid Homeostasis
Source: PLoS One. 2014 Aug 22;9(8):e105674. doi: 10.1371/journal.pone.0105674 (PMC4141819; doi:10.1371/journal.pone.0105674)
Supplement: Table S2 — A complete list of 84 genes that were profiled by qPCR using the murine Unfolded Protein Response RT2 Profiler PCR Array system, shown grouped according to their functions. (DOCX) [file pone.0105674.s004.docx]

**Table S2**

A complete list of 84 genes that were profiled by qPCR using the murine Unfolded Protein Response RT² Profiler PCR Array system, shown grouped according to their functions.

**Unfolded Protein Binding:** Calr, Canx, Cct4, Cct7, Dnajb2, Dnajb9, Dnajc10, Dnajc4, Ero1lb, Hspb9, Hspa2, Htra2, Pfdn2, Pfdn5, Ppia, Scap, Sec63, Sil1, Tcp1, Tor1a, Ugcgl1.

**ER Protein Folding Quality Control:** Edem1, Edem3, Ganab, Ganc, Prkcsh, Rpn1, Serp1, Erp44, Ugcgl1, Ugcgl2.

**Regulation of Cholesterol Metabolism:** Insig1, Insig2, Mbtps1, Mbtps2, Scap, Srebf1, Srebf2.

**Regulation of Translation:** Eif2a, Eif2ak3, Ppp1r15b, Serp1.

**ER Associated Degradation (ERAD):** Amfr, Derl1, Derl2, Edem1, Fbxo6, Herpud1, Htra2, Htra4, Mbtps1, Mbtps2, Nploc4, Nucb1, Os9, Sel1l, H47, Syvn1, Sec62, Ubxn4, Vcp.

**Ubiquitination:** Amfr, Edem3, Herpud1, Rnf139, Rnf5, Sel1l, Sec62, Ube2g2, Ube2j2, Ubxn4, Ufd1l, Usp14, Vcp.

**Transcription Factors:** Atf4, Atf6, Atxn3, Calr, Cebpb, Creb3, Creb3l3, Atf6b, Ddit3, Ern1, Ern2, Mbtps1, Pfdn5, Scap, Srebf1, Srebf2, Xbp1.

**Protein Folding:** Calr, Canx, Cct4, Cct7, Dnajb2, Dnajb9, Dnajc10, Dnajc4, Ero1l, Hspa4l, Pfdn2, Pfdn5, Ppia, Sec63, Sil1, Tcp1, Tor1a, Erp44, Ugcgl1.

**Protein Disulfide Isomerization:** Ddit3, Dnajc10, Ero1l, Ero1lb, Pdia3, H47, Srebf1, Erp44.

**Heat Shock Proteins:** Dnajb2, Dnajb9, Dnajc10, Dnajc3, Dnajc4, Hspa1l, Hspa4, Hspa5 (BiP), Hsph1, Sec63.

**Apoptosis:** Armet, Atxn3, Bax, Calr, Cebpb, Ddit3, Eif2ak3, Ern1, Ern2, Hspb9, Htra2, Mapk10, Mapk8, Mapk9, Pdia3, Ppp1r15b, H47, Vcp.
