# Supplementary material for: Cartilage-Specific Ablation of Site-1 Protease in Mice Results in the Endoplasmic Reticulum Entrapment of Type IIB Procollagen and Down-Regulation of Cholesterol and Lipid Homeostasis
Source: PLoS One. 2014 Aug 22;9(8):e105674. doi: 10.1371/journal.pone.0105674 (PMC4141819; doi:10.1371/journal.pone.0105674)
Supplement: Table S3 — A partial list of genes significantly up-regulated in S1P cko chondrocytes identified from microarray analysis. Shown are top 20 genes that are differentially up-regulated when compared to WT, and some selected genes. RNAs with no corresponding gene names are not included in this list. (DOCX) [file pone.0105674.s005.docx]

**Table S3**

A partial list of genes significantly up-regulated in S1P*^cko^* chondrocytes identified from microarray analysis. Shown are top 20 genes that are differentially up-regulated when compared to WT, and some selected genes. RNAs with no corresponding gene names are not included in this list.

| **Gene Symbol** | **Fold Change** | **Gene Name** |
| --- | --- | --- |
| Fgf21 | 53.2457 | Fibroblast growth factor 21 |
| Otog | 33.5656 | Otogelin |
| Trib3 (Nipk) | 28.2282 | Tribbles homolog 3 |
| Fibcd1 | 26.1165 | Fibrinogen C domain containing 1 |
| Cdsn | 19.6674 | Corneodesmosin |
| Avil | 19.0096 | Advillin |
| Slc7a3 | 15.1412 | Solute carrier family 7 |
| Cxadr | 9.24044 | Coxsackie virus and adenovirus receptor |
| Atf4 | 8.96989 | Activating transcription factor 4 |
| Chac1 | 8.25859 | Cation transport regulator-like protein 1 |
| Ddit3 | 7.85657 | DNA damage inducible transcript 3 |
| B4galnt2 | 7.36378 | Beta-1,4-N-acetyl-galactosaminyl transferase 2 |
| Inhbe | 6.59675 | Inhibin, beta E |
| Gpnmb | 6.05538 | Glycoprotein (transmembrane) nmb |
| Slc6a9 | 5.57524 | Solute carrier family 6 |
| Angptl6 | 5.18552 | Angiopoietin-like 6 |
| Cyb5r2 | 4.89807 | Cytochrome B5 reductase 2 |
| LOC100047619 | 4.70166 | Similar to solute carrier family 7 |
| SSPO | 4.48232 | SCO-spondin |
| Nupr1 (Protein p8) | 4.1821 | Nuclear protein, transcriptional regulator 1 |
| IBSP | 4.16453 | Integrin binding sialoprotein |
| Gch1 | 3.35972 | GTP cyclohydrolase 1 |
| Asns | 3.31098 | Asparagine synthetase |
| Atf5 | 3.22215 | Activating transcription factor 5 |
| Gpt2 | 3.18373 | Glutamic pyruvate transaminase (alanine aminotransferase) 2 |
| Hap1 (Apex) | 2.81318 | DNA apurinic/apyrimidinic (AP) endonuclease |
| Eif4ebp1 | 2.54603 | Eukaryotic translation initiator factor 4E binding protein 1 |
| Sars | 2.5227 | Seryl t-RNA synthetase |
| Cdkn1a (p21) | 2.30742 | Cyclin dependent kinase inhibitor 1a |
| Mll1 | 2.09924 | Myeloid/lymphoid leukemia |
