# Supplementary material for: Cartilage-Specific Ablation of Site-1 Protease in Mice Results in the Endoplasmic Reticulum Entrapment of Type IIB Procollagen and Down-Regulation of Cholesterol and Lipid Homeostasis
Source: PLoS One. 2014 Aug 22;9(8):e105674. doi: 10.1371/journal.pone.0105674 (PMC4141819; doi:10.1371/journal.pone.0105674)
Supplement: Table S4 — A partial list of genes significantly down-regulated in S1P cko chondrocytes identified from microarray analysis. Shown are top 20 genes that are differentially down-regulated when compared to WT, and some selected genes that belong to fatty acid and cholesterol biosynthesis pathways. RNAs with no corresponding gene names are not included in this list. (DOCX) [file pone.0105674.s006.docx]

**Table S4**A partial list of genes significantly down-regulated in S1P*^cko^* chondrocytes identified from microarray analysis. Shown are top 20 genes that are differentially down-regulated when compared to WT, and some selected genes that belong to fatty acid and cholesterol biosynthesis pathways. RNAs with no corresponding gene names are not included in this list.

| **Gene Symbol** | **Fold Change** | **Gene Name** |
| --- | --- | --- |
| Fgfr1op2 | -12.4085 | Fibroblast growth factor receptor 1 oncogene partner 2 |
| Sparc | -11.2739 | Osteonectin (secreted protein, acidic, cysteine-rich) |
| Krtap13 | -11.0803 | Keratin associated protein 13 |
| Lor | -9.17809 | Loricrin |
| Lce1m | -7.22621 | Late cornified envelope protein 1m (small proline-rich protein) |
| Prg4 | -6.49905 | Proteoglycan 4 (lubricin) |
| Scd1 | -6.09877 | Stearoyl-CoA-desaturase 1 |
| Crct1 | -5.41591 | Cysteine-rich C-terminal 1 |
| Lce1a1 | -4.99055 | Late cornified envelope protein 1A1 |
| Tm7SF3 | -4.53791 | Transmembrane 7 superfamily member 3 |
| C1qtnf3 | -4.53193 | Complement C1q tumor necrosis factor related protein 3 |
| Lce3F | -4.24526 | Late cornified envelope protein 3F |
| Ldlr | -4.16262 | Low-density lipoprotein receptor |
| Kprp | -3.89984 | Keratinocyte proline-rich protein |
| Ppp1r3b | -3.85025 | Serine threonine phosphatase, protein phosphatase 1 |
| Sc4mol | -3.75594 | Sterol-C4-methyl oxidase-like |
| Pcsk9 | -3.74258 | Proprotein convertase subtilisin/kexin type 9 |
| Insig1 | -3.62052 | Insulin-induced gene 1 |
| Calm4 | -3.60394 | Calmodulin 4 |
| Stard4 | -3.13912 | Star-related lipid transfer (START) domain containing 4 (Stard4) |
| Fads2 | -3.05881 | Fatty acid desaturase 2 |
| Lss | -2.77893 | Lanosterol synthase |
| Fdps | -2.66886 | Farnesyl diphosphate synthase |
| Hmgcr (Hmdh) | -2.50716 | 3-Hydroxy-3-methylglutaryl-CoA reductase |
| Idh1 | -2.28061 | Isocitrate dehydrogenase 1 |
| Scd2 | -2.21651 | Stearoyl-CoA-desaturase 2 |
| Nsdhl | -2.15225 | NAD(P) dependent steroid dehydrogenase like gene |
| Hapln3 | -2.06203 | Hyaluronan and proteoglycan link protein 3 |
| Sqle (Erg1) | -2.14067 | Squalene epoxidase gene |
| Cyp51 | -2.01021 | Sterol 14-alpha-demethylase |
